# Understanding the ability of households to cope with economic shocks: an empirical study of Pakistan during the COVID-19 pandemic

**DOI:** 10.1186/s41043-025-00772-y

**Published:** 2025-03-19

**Authors:** Arslan Austin, Imran Ur Rahman, Zunera Rana

**Affiliations:** 1 Faculty of Communication and Environment, Rhine-Waal University of Applied Sciences, Friedrich-Heinrich-Allee 25, 47475 Kamp-Lintfort, Germany; 2 Center for Trans-Himalaya Studies, Leshan Normal University, Leshan, China; 3 Department of Anthropology and Development Studies, Faculty of Social Sciences, Radboud University, Nijmegen, The Netherlands

**Keywords:** COVID-19, Pakistan, Coping, Lower-middle income country, Household

## Abstract

**Background:**

The COVID-19 pandemic presented unprecedented challenges to households throughout the world, particularly in low- and middle-income countries. Pakistan’s COVID-19 management policies have been widely recognized for their effectiveness at both national and international levels.

**Objective:**

In this study, we empirically examine households’ response to external shocks, such as the COVID-19 pandemic, and the coping mechanisms adopted at the household level in Pakistan.

**Methods:**

Based on Rational Choice Theory, the research examines 3456 households, encompassing both urban and rural areas, using official survey data from the National Bureau of Statistics of Pakistan. The study utilizes the logit model for the estimations.

**Results:**

The findings show that substitution for low-quality food sources is the most common coping mechanism and closely impacts food security. Interestingly, the study revealed that, except for bank loans, none of the coping mechanisms significantly reduced the likelihood that families would experience severe COVID-19 effects.

**Conclusions:**

The findings of the study underscore the complexities of responding to a multifaceted crisis such as the pandemic. This research contributes essential insights into the evolving discourse on pandemic resilience, recovery strategies, and anticipated similar shocks.

## Introduction

The coronavirus pandemic has caused significant socioeconomic disruptions worldwide and highlighted the vulnerabilities within health systems and the lack of preparedness at the global level to address external and internal shocks to economies. This has been especially true for low- and lower middle-income countries like Pakistan, which were already struggling to keep up with high-income economics due to instability, climate vulnerabilities, limited resources, poor governance and a lack of capacity for immediate policy response. The sudden and severe nature of the pandemic created unprecedented challenges for families, ranging from income losses to restricted access to essential services, balance of payment crisis [1], increasing budget deficits [2], high inflation rates and rising unemployment rates [3], which have been exacerbated further due to supply chain disruptions. Understanding how households respond to such external shocks is crucial for formulating targeted and effective policy interventions.

**Fig. 1:**
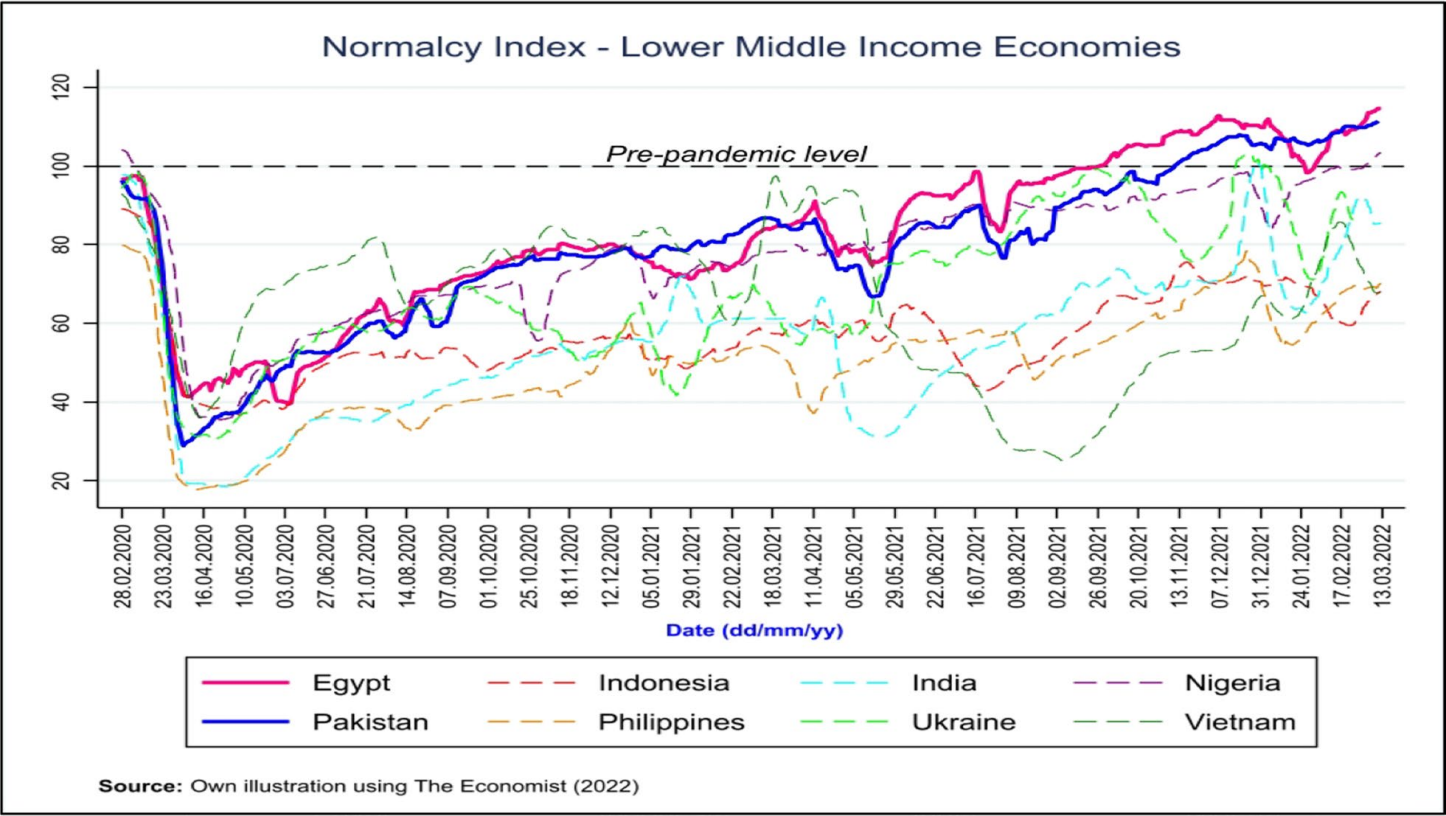
Normalcy Index-lower middle-income economies

While governments of low- and lower middle-income countries have struggled to survive during the pandemic, this struggle has been far worse at the household level. According to estimates by the World Bank (2021), more than half a billion people were pushed into extreme poverty during the initial year of the pandemic, mostly because of increased health costs, the loss of jobs and the lack of a social security system to protect them [4]. Similarly, school enrollment rates in low- and lower middle-income countries (for example, Pakistan) have also shown a downward trend during the pandemic, which has been coupled with a lack of facilities for home schooling and distance learning at the household level [5]. According to the normalcy index developed by Economist (2022), most low middle-income countries are still struggling to reach pre-pandemic levels of normalcy even two years after the pandemic (see Fig. 1) [6].

Overall, research on the coronavirus pandemic has been quite diverse. Some researchers have analyzed the impact of the pandemic at the micro level by examining health systems in both high-income and low-income countries [7], whereas others have observed the influence of lockdown policies on industries, including SMEs [8, 9], global supply chains [10] and the responses of different countries [11, 12]. However, other researchers have examined macrolevel impacts by examining the global response of the world to the pandemic [13–15].

Within the development literature, there has been increased discussion on the effects of lockdown policies on demographic indicators in developing countries [16, 17]. However, there has been less discussion on the impact of coronavirus lockdown policies at the household level and the coping strategies that households have utilized to overcome the struggles associated with extended lockdowns. We believe that studying these coping strategies can help shape policy implications for future pandemics. At the same time, it can also allow both governments and international donors within the development sector to understand the vulnerabilities of households in the development of effective pandemic mitigation strategies.

The main question that we attempt to answer through our research is as follows: What coping strategies were used by severely marginalized households during the coronavirus disease 2019 (COVID-19) lockdown to mitigate its impact on household income? To answer our research question, we base our study methodology on the rational choice theory. We use empirical modeling techniques, including binary regression models and marginal effects, as post estimates for a sample of 3456 representative household surveys. We utilize binary logistic model to estimate our results, as our main dependent variable assumes binary values (0, 1).

To make our analysis more profound, we focus on only one country, i.e., Pakistan. We have chosen Pakistan as our country of analysis for several reasons. First, Pakistan has been applauded for its effective COVID-19 management policies, both nationally and internationally [18]. Second, it has been among the few countries that did not opt for a complete lockdown [17]. Third, the country has been struggling with high poverty rates, poor health infrastructure and increasing population rates, which has led to challenges in terms of developing and implementing an effective coronavirus policy. Therefore, the impact of the measures and the resilience of households in navigating economic hardships remain areas that require deeper exploration.

Despite these challenges, Pakistan has been among the few lower middle-income countries that are able to return to normal, as shown in Fig. 1. For these reasons, Pakistan is an interesting case study for analyzing the household impact of coronavirus disease 2019 (COVID-19) lockdown measures.

By analyzing coping strategies and their effectiveness across different socioeconomic and geographic contexts, this research seeks to provide valuable insights into the factors influencing household resilience. Our study adds to the current literature in several ways. First, very few studies have empirically examined the household impact of the coronavirus pandemic and the coping strategies of these households. To the best of our knowledge, there are no such studies for Pakistan that specifically use this dataset. Moreover, our study provides policy guidelines for pandemic management based on empirical evidence, which is missing from the literature.

We continue our paper as follows: In the next section, we review the current literature on this topic. In Sect. "Pandemic response in Pakistan", we provide a brief overview of the pandemic situation in Pakistan and the relevant government policies. This is followed by Sect. "Methodology", where we discuss the methodology of our paper. In Sects. "Results" and "Discussion of results and implications", we analyze the results of our empirical model and explain the policy implications for low- and lower-middle-income countries. Finally, in Sect. "Conclusion", we conclude our paper.

## Literature review

The COVID-19 pandemic has had far-reaching consequences for economies and societies worldwide, with the global economy shrinking by 4.3% in 2020 [19]. In addition to health-related problems, the pandemic has had diverse economic costs. This included significant disruptions to businesses and supply chains, which led to job losses (both in formal and informal labor markets) and reduced incomes [20, 21].

Moreover, the pandemic has resulted in social consequences, including increased food insecurity, reduced access to healthcare services, and disrupted education, especially in low- and lower-middle-income countries [7]. Many households in these countries face the tough choice of spending on health or food as sources of income depleted. Analyzing the impact of COVID-19 in Uganda, Ethiopia, Nigeria and Malawi, Josephson et al. (2021) estimated that approximately 256 million people were living in households that have suffered severe income losses. This was due to government pandemic policies in which an estimated 36 million adults suffered from food insecurity. It was also reported that the impact on income losses was similar in rural and urban areas in these countries, whereas problems related to food security were greater in poorer households [7, 22].

Yue et al. (2020) reported a change in the behavior of households due to uncertainties resulting from the pandemic [23]. They concluded that households that have been impacted by COVID-19 are more likely to change their risk behavior and become risk averse. This would imply that the households were less likely to invest in risky portfolios or take on additional debt.

Households in low- and lower-middle-income economies usually lack social welfare systems to fall back on in the case of external shocks such as the pandemic. Many of these households rely on family networks and a combination of a decrease in income and an increase in informal borrowing to cope in the absence of continuous income [24]. Studies also identified several coping strategies resulting from external shocks in Latin American households, including increasing the number of people working per household (for example, incorporating women into the labor force), adjusting consumption patterns for education, health and clothing, participating in networks of mutual assistance and changing the composition of households [24]. While these mechanisms allow households to survive external shocks, they also significantly impact the level of inequality in society.

Household coping strategies also depend on the gender of the head of the family. According to a study based on Kenya and Uganda, female-headed households are less likely to use asset selling or taking loans as a coping mechanism than male-headed households. Female-headed households rely more on social networks to cope, including friends and family [25].

Similarly, some researchers do not regard COVID-19 as an idiosyncratic or individual-level hazard but rather as a community-level shock [26]. Given its effects on the community level as well as on all community linkages to regional, national, or global levels, it may be argued that it is a strong community shock. The scope of this epidemic raises significant concerns regarding the effectiveness of typical coping and adaptation techniques, such as livelihood diversification [27]. Investigating and identifying the main coping mechanisms used by families in the context of the pandemic is therefore crucial. This study attempts to fill this gap in the context of Pakistan.

Some researchers have also shed light on the economic and social repercussions that the COVID-19 pandemic has had in several nations throughout the world [20–22]. However, studies on mitigation techniques that focus on the COVID-19 pandemic in lower-middle income countries are lacking and require in depth investigation.

Overall, the literature discusses the impact of the pandemic at the global and individual levels and discusses the coping strategies used by households to ensure their survival in the case of an external shock. However, an analysis of coping strategies among households in South Asia is lacking in the literature. Therefore, we hope to add to the current literature by analyzing the case of Pakistan. Given that we have provided an overview of the literature, we provide a brief overview of Pakistan’s response to COVID-19 and its implications at the household level.

## Pandemic response in Pakistan

According to the labor force survey of Pakistan, in 2020–21, almost 74% of its labor force was employed in the informal economy [28]. Moreover, almost 40% of workers receive payments in cash either daily or weekly, proxying for daily wage earners. Between 2004 and 2018, the national poverty level of the country decreased from 50 to almost 20% [4]. However, decreasing levels of poverty have been negatively affected by the pandemic, as poverty is estimated to return to 40% by 2024 [29]. This could lead to food insecurity, an increase in debt burden and an immediate need for safety nets and community support. As of January 2023, inflation in Pakistan stood at 27.55% [30], the GDP growth rate has been predicted to be approximately 0.3% [31], and unemployment rates have been calculated as 8.5% [32]. It is estimated that almost 56.6% of the population will become vulnerable according to the multidimensional vulnerability index in 2022 as a result of the pandemic [28]. This vulnerability has a major impact on 64% of the youth population of Pakistan [33], who are already facing a lack of employment opportunities, and the sudden shock and restrictions amid the COVID-19 pandemic have further deteriorated these conditions.

In the face of several moving targets, a balancing act between public health and socioeconomic consequences led to the most epic dilemma for Pakistan’s government in terms of whether to fully lock down its economy. This necessity mothered a selective nonpharmaceutical intervention known as *smart lockdown*. This method allows for containing the disease under strict restrictions within high infection areas and continuing with less restricted measures in outer zones [34]. This strategy inspired other countries to follow the example of Pakistan [35]. These smart lockdowns were also relevant to avoid a total collapse of already crippling health infrastructure and already existing infestations concerning dengue, malaria and poliovirus [36].

Pakistan was hit consistently from April 3, 2020, to February 23, 2022, with five different waves separated by different COVID-19 variants [37]. There were almost 1.5 million cases recorded, with a 2% death rate of almost 28 thousand people. Pakistan ranks 35th in terms of total deaths and 53rd in terms of total cases [38]. This relatively less worsened outcome is attributed to the strategy of smart lockdown. In addition to smart lockdowns, the government of Pakistan approved a financial stimulus of Rs 1.2 trillion and another complementary grant of Rs.100 billion to address the impact of COVID-19 [39].

Monetary policy rates were cut from 13.25 to 7% from March to June, indicating that it was the largest reduction in interest rates among the emerging economies to address the pandemic [40]. The commercial loans amounting to Rs. 910 billion were deferred by the State Bank of Pakistan, Rs. 717 billion for the commercial sector, Rs. 27 billion for SMEs, Rs. 43 billion for private financing and Rs. 121 billion for microfinancing banks [40, 41]. Safety net tools for social assistance, such as Zakat, Pakistan Bait-ul-mal, BISP (Benazir Income Support Program) and the Ehsas program, were further financed to protect the existing and newly impacted most vulnerable individuals [42]. The prime minister capitalizing on his goodwill with Pakistani citizens abroad announced a prime minister’s corona relief fund that activated a donation-based inflow of remittances [43].

Pakistan faced a V-shaped economic recovery in 2022, with economic growth rates of 5.74% in 2021 and 5.97% in 2022. However, in the summation of goods and bads of these policies, economic growth was deemed unsustainable given the financial and macroeconomic imbalances [44]. Against this background, it is imperative to study the impacts of the diverse measures on the coping strategies of households in different areas of Pakistan. In addition to these measures, we want to assess the trickledown impact of such policy efforts on households in Pakistan, both in rural and urban areas. We discuss the theoretical approach of this trickledown effect in more detail in the next section.

## Methodology

### Theoretical approach

The theoretical underpinning of our empirical model is based on rational choice theory, which uses an optimal (satisfying bounded rationality)^1^ consumption bundle at the household level for income and the substitution effect for the adaptation of coping strategies of households because of the pandemic [45, 46].

Rational choice theory is a neoclassical framework for defining the optimization function of microeconomic agents in the face of constraints such as given budget levels and information sets [45]. In the case of a change in budget constraints, rational consumers usually opt for two strategies: they either change their consumption patterns (also known as the income effect) or replace cheaper/expensive goods with expensive/cheaper goods, respectively (called the substitution effect), as their marginal propensity to consume changes.

Since the discussion in Sects. "Literature review" and "Pandemic response in Pakistan", it is safe to assume that external shocks such as the pandemic may cause changes in household income (budget) and thus lead to changes in the choices of consumption patterns (preferences). Therefore, we attempt to investigate, because of the pandemic, whether there was a change in the budget (income effect due to loss of employment) or the preferences (substitution effect due to medication or prices of available goods) of the households.

The investigation of income and substitution effects in coping methods may yield useful insights into the prioritization of family spending and the trade-offs made during periods of crisis. We discuss this in greater detail as we analyze the results from our empirical model. In the next section, we explain the empirical model.

### Empirical approach

We employ the empirical approach of working with binary models. Based on the information criteria and robustness checks,^2^ we chose the logit model over the probit model. The justification for choosing one model over the other remains rather theoretical and, in practice, has rare differences other than mathematical convenience [48]. Following Greene (2012) [48], a logit model assumes a logistic probability function as follows with a cumulative distribution function denoted as $\Lambda (.)$:

$${\text{Prob}} ({\text{Y}} = 1 | {\text{x}}) = \frac{{\exp \left( {x^{\prime } \beta } \right)}}{{1 + \exp \left( {x^{\prime } \beta } \right)}} = \Lambda \left( {x^{\prime } \beta } \right)$$

For interpretation, marginal effects as post estimates are measured, for example, for an independent variable d.

$${\text{Marginal effect}} = {\text{Prob}}\left[ {{\text{Y}} = 1\left| {\overline{x}_{{{\text{(d}})}} } \right. , {\text{d}} = 1 - {\text{Prob}}} \right]\left[ {{\text{Y}} = 1 \left| {\overline{x}_{{\left( {\text{d}} \right)}} } \right. {\text{d}} = 0} \right]$$

The marginal effects explained in the equation above measure one unit change in the independent variable (0 to 1), leading to a change in the probability of the dependent variable. We measure a full model including all the variables listed in Table 1 along with divisions across rural and urban settings and provincial models (Appendix 1). For the policy implications and robustness checks, we deem it necessary to also consider the potential substitutive effect between different coping strategies (Appendix 3).

**Table 1:** Description of variables

Variable	Description	Measurement
*Dependent variable*	How severely has your household been affected by the Covid-19?	
HHaffected	Not at all affected	1 if highly affected and severely affected 0 otherwise
	Mildly affected	
	Moderately affected	
	Highly affected	
	Severely affected	
Independent variable	What the household did to cope up the economic situation during COVID-19?	
*Food security*		
Foodreduction	Reduced quantity of food intake	1 if Yes
Lowqualityfood	Switched to lower quality or cheaper food	0 if No
*Debt burden*		
Loanrelatives	Loans from relatives/friends	1 if Yes 0 if No
Loanemployer	Loans from employer/moneylenders/traders	
Loanbanks	Loans from formal sources/NGOs/Banks	
Communityassistance	Asked and received help/gift assistance from others in the community (not loans)	
Loandeferment	Delayed payment of loans	
*Energy poverty*		
Electricitydefault	Nonpayment of Electricity bills	1 if Yes 0 if No
Gasdefault	Nonpayment of Gas bills	
*Essentials*		
Nonfoodreduction	Reduced nonfood expenses, i.e., health and education, clothing/shoes etc	1 if Yes 0 if No
Schooldropout	Discontinuation of Education of children due to nonavailability of monthly fee	
Displacement	Temporary Migration due to loss of job/Migrated to look for livelihood opportunities	
*Capital flight*		
Productiveassets	Sold productive assets or means of transport (sewing machine, wheelbarrow, grain mill, agricultural tools, farm machinery, bicycle, car etc.)	1 if Yes 0 if No
Savings	Spent saving or investments	
HHassets	Sold household assets/goods (radio, furniture, refrigerator, television, jewelry etc.)	
Productivecattle	Sold last productive/female animal	
Stockseed	Consumed seed stock held for the next season	
Soldland	Sold house/land/plot	
*Controls*		
YoungHH	Household Roster: Age	1 if average age of household member is > 24 and 0 otherwise
	(In completed years on last Birthday)	1if average gender balance is > 0.5 and 0 otherwise
	Household Roster: Sex	1 if at least 20% of the household members have school education
MaledominatedHH	Male Female	0 otherwise
EducatedHH	Household Roster: Maximum Education attained	1 if ≥ 25,000 PKR
AboveavgHHincome	Section B – impact of COVID-19 on employment and income: Average Monthly income during Covid-19?	0 Otherwise
Remittances	Section B – impact of COVID-19 on employment and income: Does any member of your Household received Foreign Remittances (Rs outside Pakistan)?	1 if yes before and during the COVID-19 and 0 otherwise
Charity	Section B – impact of COVID-19 on employment and income: Does any member of your household usually receive zakat, usher, Sadaqat, or gift (kind, Cash) Including BISP, Ehsaas, Bait ul Mal public, Private	1 if yes during the COVID-19 and 0 otherwise
RuralHH	Control	Yes
Provincial fixed effects	Control	Yes

### Data and variables

To address our research question, we employ the official dataset published by the Pakistan Bureau of Statistics as a result of a nationwide survey conducted electronically from the 20th of October 2020, to the 5th of November 2020 [49]. The survey utilized is representative of provinces, the federal capital, Gilgit Baltistan and Azad Jammu and Kashmir. Furthermore, the sampling technique counts enumeration blocks/villages as primary sampling units while considering stratification at the administrative division and district, rural and urban levels. In total, 500 sampling blocks are defined, and 6000 households are interviewed following a structured survey. Our final sample is based on 3456 households after the use of merging techniques. The purpose of the survey is to formulate informed policies with a specific focus on employment, food security and the general well-being of the population at large. To the best of our knowledge, no other study has utilized this unique dataset.

Our dependent variable is created from the survey question: “How severely has your household been affected by COVID-19?”. In line with our research question, to focus on households that were severely marginalized during the COVID-19 shock, we categorize ‘highly’ and ‘severely’ as ‘1’, while the remaining variations are categorized as ‘0’. On the basis of the variables discussed in Sect. "Literature review", we analyze the impacts of food security, debt burden, energy poverty, household essentials, and capital flight. Furthermore, control variables on average household age, education, income, male gender dominance, remittances and ‘zakat^3^’ and other government subsidy programs as a mode of charity and safety net have been added along with subregional fixed effects. Table 1 provides a complete list of variables and their descriptions.

## Results

In Table 2, in the second column, we present descriptive statistics for our full model consisting of all the households at the national level of our sample. The next columns show the models that consider the comparative impact of COVID-19 on rural households and urban households. To emphasize this, we measure the delta difference between urban and rural households. We also divide the data at the provincial level and include descriptive statistics related to it in Tables 6, 7, 8, 9, 10, and of Appendix 1.

**Table 2:** Descriptive statistics

Variable	Full Model (n = 3456)	Rural Model (n = 1036)	Urban Model (n = 2420)	Delta (Urban – Rural)
AffectedHH	0.280 (0.449)	0.268 (0.443)	0.284 (0.451)	0.016
*Food security*				
Foodreduction	0.698 (0.459)	0.712 (0.453)	0.692 (0.462)	− 0.02
Lowqualityfood	0.754 (0.431)	0.764 (0.425)	0.749 (0.434)	− 0.015
*Debt burden*				
Loanrelatives	0.443 (0.497)	0.401 (0.490)	0.461 (0.499)	0.06
Loanemployer	0.120 (0.325)	0.149 (0.356)	0.107 (0.310)	− 0.042
Loanbanks	0.045 (0.208)	0.035 (0.183)	0.050 (0.218)	0.015
Communityassistance	0.128 (0.334)	0.095 (0.293)	0.142 (0.349)	0.047
Loandeferment	0.218 (0.413)	0.191 (0.393)	0.230 (0.421)	0.039
*Energy poverty*				
Electricitydefault	0.404 (0.491)	0.375 (0.484)	0.417 (0.493)	0.042
Gasdefault	0.269 (0.443)	0.179 (0.383)	0.307 (0.462)	0.128
*Essentials*				
Nonfoodreduction	0.839 (0.367)	0.810 (0.393)	0.852 (0.355)	0.042
Schooldropout	0.168 (0.374)	0.102 (0.303)	0.196 (0.397)	0.094
Displacement	0.035 (0.184)	0.030 (0.170)	0.037 (0.189)	0.007
*Capital flight*				
Productiveassets	0.057 (0.232)	0.059 (0.236)	0.056 (0.230)	− 0.003
Savings	0.723 (0.448)	0.618 (0.486)	0.767 (0.423)	0.149
HHassets	0.077 (0.266)	0.058 (0.234)	0.085 (0.279)	0.027
Productivecattle	0.083 (0.276)	0.172 (0.377)	0.045 (0.207)	− 0.127
Stockseed	0.024 (0.154)	0.045 (0.208)	0.015 (0.123)	− 0.03
Soldland	0.010 (0.100)	0.008 (0.088)	0.011 (0.105)	0.003
*Controls*				
YoungHH	0.503 (0.500)	0.481 (0.500)	0.513 (0.500)	0.032
MaledominatedHH	0.679 (0.467)	0.656 (0.475)	0.688 (0.463)	0.032
EducatedHH	0.364 (0.481)	0.346 (0.476)	0.372 (0.483)	0.026
AboveavgHHincome	0.401 (0.490)	0.355 (0.479)	0.421 (0.494)	0.066
Remittances	0.034 (0.181)	0.031 (0.173)	0.035 (0.184)	0.004
Charity	0.401 (0.490)	0.384 (0.487)	0.408 (0.492)	0.024
RuralHH	0.300 (0.458)			
KP	0.131 (0.337)			
Punjab	0.318 (0.466)			
Sindh	0.326 (0.469)			
Balochistan	0.120 (0.325)			

The standard deviations are in parentheses. All the variables in the three models have max = 1.000 and min = 0.000

As shown in Table 2, a comparable level of household impact across regional heterogeneity can be inferred due to the pandemic: 28% in both the full model and urban areas and 26.8% in rural areas. The most prevalent coping strategy is estimated to be nonfood reduction, which may be because it includes several other factors that are nonfood related, such as health, education, and clothing. This is followed by the substitution of low-quality food by almost 75% of the households across the three fractions. This set food security as the primary or immediately impacted category due to COVID-19, which is in line with the literature, as discussed in Sect. "Literature review" [50, 51].

In the third position, burning into the savings of the household appears as a coping strategy, with 72% in the full model, 77% in urban Pakistan and 62% in rural Pakistan, with delta = 15%. This is also the largest differentiating coping strategy between rural and urban households in our sample. The underlying explanations are somewhat clear; it can be perceived that rural households have less savings in monetary terms and more so in terms of productive cattle and stock seeds (delta = $-$ 13% and $-$ 3%, respectively).

The next highest savings are gas defaults, with delta = 13%, possibly because rural Pakistan has far less access to gas connections [52]. Additionally, a staggering 40% of households across the three models defaulted on their energy bills, indicating that energy poverty resulted from the COVID-19 crisis in Pakistan. Taking loans from relatives and deferring loan payments turns out to be the coping strategy to manage the debt burden. Asking for community assistance or loans from employers is also used, although loans from employers are predicted to be more common in rural areas than in urban areas (delta = 4.2%). The statistics also show that loans from banks are the least adapted coping strategy, perhaps because of the dwindling creditworthiness of borrowers, financial exclusion, and the COVID-19 pandemic struggling with the banking sector of Pakistan [53, 54].

In terms of school dropout in the essential category, 20% of urban households had to quit sending their children to school compared with 10% of households in rural areas; across Pakistan, this predicted number was 17%. This highlights an alarming blowback for one of the lowest schools with children and the lowest literacy rate in the region [4]. Displacement also slightly accounted for 3.5%, more so in urban households than in rural households, most likely because of economic activity [55].

According to the estimates, approximately 5% to 7% of households had to sell productive or household assets, whereas only 1% had to sell land. Households are somewhat equally distributed in terms of their age, while approximately 68% of households are male dominated according to the statistics. Compared with rural households, 40% of Pakistani households have above average income, whereas urban households have a higher average income (delta = 6%), perhaps because urban households are also marginally more educated (delta = 2%). Finally, charity has cushioned the impact of COVID-19 in 40% of households across Pakistan, which is slightly greater for urban households than for rural households. Remittances accounted for only 3-4% across the three models.

In Tables 12, 13 and 14 in Appendix 2, we present our correlation matrices for the full model and the rural and urban models, respectively. There are no strong correlations reported. A moderate positive correlation between food reduction and low-quality food as well as between two energy poverty variables can be observed in all three tables. However, none of the correlations show signs of multicollinearity. The variance inflation factors for all the variables in all three models were also less than 5.

### Main results

In Table 3, we have estimated the logit model, whereas in Table 4, we have the marginal effects as the post estimates. Interestingly, several consistencies and discrepancies across the three models lead to valuable insights. Most importantly, our results indicate that none of the coping strategies, except for banking loans, decreased the probability of becoming severely affected by the COVID-19 pandemic. Households adapted several coping strategies in line with the literature, perhaps only to cushion against the burden of the pandemic and provide important evidence of household resilience [24, 56, 57]. Thus, the estimated marginal effects are small. Perhaps, in line with other studies, our results also provide significant evidence in favor of the ‘smart lockdown strategy’ pioneered by the federal government of Pakistan during the pandemic that time (see section "Pandemic response in Pakistan" for more details) [58]. Our results can be further checked for robustness and context in the discussion and implications section.

**Table 3:** Logit estimates

	(1)	(2)	(3)
Variables	Full model	Rural Pakistan	Urban Pakistan
*Food security*			
Foodreduction	0.956***	1.104***	0.947***
	(0.142)	(0.282)	(0.168)
Lowqualityfood	1.435***	1.241***	1.517***
	(0.196)	(0.408)	(0.227)
*Debt Burden*			
Loanrelatives	0.996***	1.103***	0.986***
	(0.0967)	(0.198)	(0.113)
Loanemployer	0.253*	0.444*	0.105
	(0.144)	(0.258)	(0.182)
Loanbanks	− 1.128***	− 0.939*	− 1.111***
	(0.233)	(0.492)	(0.272)
Communityassistance	0.576***	0.909***	0.548***
	(0.135)	(0.313)	(0.152)
Loandeferment	0.531***	0.262	0.506***
	(0.111)	(0.240)	(0.129)
*Energy poverty*			
Electricitydefault	0.385***	0.955***	0.201
	(0.117)	(0.218)	(0.146)
Gasdefault	0.0182	− 0.463	0.152
	(0.132)	(0.307)	(0.153)
*Essentials*			
Nonfoodreduction	0.297	0.166	0.342
	(0.181)	(0.346)	(0.216)
Schooldropout	0.0796	0.467	0.0695
	(0.123)	(0.295)	(0.138)
Displacement	0.450**	− 0.163	0.557**
	(0.228)	(0.531)	(0.256)
*Capital Flight*			
Productiveassets	0.248	0.218	0.234
	(0.188)	(0.384)	(0.223)
Savings	0.103	0.605***	− 0.00979
	(0.108)	(0.210)	(0.132)
HHassets	0.498***	-0.191	0.625***
	(0.162)	(0.375)	(0.183)
Productivecattle	0.236	0.298	0.290
	(0.165)	(0.240)	(0.252)
Stockseed	0.567*	0.229	0.678
	(0.295)	(0.428)	(0.450)
Soldland	-0.236	1.098	− 0.470
	(0.527)	(1.102)	(0.591)
*Controls*			
YoungHH	0.148*	0.389**	0.0831
	(0.0900)	(0.181)	(0.106)
MaledominatedHH	0.0887	0.117	0.101
	(0.0961)	(0.188)	(0.114)
EducatedHH	0.0428	0.122	− 0.00235
	(0.0938)	(0.191)	(0.110)
AboveavgHHincome	0.0253	0.341*	− 0.125
	(0.0918)	(0.190)	(0.108)
Remittances	0.281	− 0.0277	0.364
	(0.248)	(0.523)	(0.290)
Charity	0.132	0.0471	0.131
	(0.0911)	(0.187)	(0.106)
RuralHH	− 0.0531		
	(0.105)		
Constant	− 3.743***	− 4.601***	-3.509***
	(0.269)	(0.488)	(0.325)
Regional FE	Yes	Yes	Yes
Observations	3,456	1,036	2,420
Pseudo R2	0.239	0.317	0.224
chi2	979.5	381.6	648.4
Log-likelihood	− 1558	− 411.7	− 1120

Standard errors in parentheses ***p < 0.01, **p < 0.05, *p < 0.1

**Table 4:** Logit marginal effects

	(1)	(2)	(3)
Variables	Full model	Rural Pakistan	Urban Pakistan
*Food Security*			
Foodreduction	0.141***	0.140***	0.145***
	(0.0206)	(0.0349)	(0.0251)
Lowqualityfood	0.212***	0.158***	0.232***
	(0.0285)	(0.0513)	(0.0341)
*Debt Burden*			
Loanrelatives	0.147***	0.140***	0.151***
	(0.0135)	(0.0238)	(0.0163)
Loanemployer	0.0374*	0.0564*	0.0161
	(0.0212)	(0.0326)	(0.0279)
Loanbanks	− 0.167***	− 0.119*	− 0.170***
	(0.0340)	(0.0621)	(0.0410)
Communityassistance	0.0851***	0.115***	0.0838***
	(0.0197)	(0.0391)	(0.0230)
Loandeferment	0.0785***	0.0333	0.0774***
	(0.0162)	(0.0304)	(0.0195)
*Energy Poverty*			
Electricitydefault	0.0569***	0.121***	0.0308
	(0.0172)	(0.0267)	(0.0222)
Gasdefault	0.00269	− 0.0588	0.0233
	(0.0196)	(0.0389)	(0.0233)
*Essentials*			
Nonfoodreduction	0.0439	0.0210	0.0523
	(0.0267)	(0.0439)	(0.0331)
Schooldropout	0.0118	0.0594	0.0106
	(0.0182)	(0.0374)	(0.0210)
Displacement	0.0665**	− 0.0207	0.0852**
	(0.0336)	(0.0675)	(0.0390)
*Capital Flight*			
Productiveassets	0.0366	0.0277	0.0358
	(0.0278)	(0.0487)	(0.0341)
Savings	0.0152	0.0768***	− 0.00150
	(0.0160)	(0.0264)	(0.0202)
HHassets	0.0737***	− 0.0242	0.0955***
	(0.0238)	(0.0476)	(0.0277)
Productivecattle	0.0349	0.0379	0.0443
	(0.0244)	(0.0304)	(0.0386)
Stockseed	0.0839*	0.0292	0.104
	(0.0435)	(0.0543)	(0.0687)
Soldland	− 0.0348	0.140	− 0.0719
	(0.0779)	(0.140)	(0.0903)
*Controls*			
YoungHH	0.0219*	0.0495**	0.0127
	(0.0133)	(0.0228)	(0.0162)
MaledominatedHH	0.0131	0.0148	0.0155
	(0.0142)	(0.0239)	(0.0175)
EducatedHH	0.00633	0.0155	− 0.000359
	(0.0139)	(0.0242)	(0.0169)
AboveavgHHincome	0.00374	0.0433*	− 0.0191
	(0.0136)	(0.0240)	(0.0164)
Remittances	0.0415	-0.00352	0.0556
	(0.0366)	(0.0665)	(0.0442)
Charity	0.0196	0.00598	0.0201
	(0.0135)	(0.0237)	(0.0163)
RuralHH	− 0.00784		
	(0.0155)		
Regional FE	Yes	Yes	Yes
Observations	3,456	1,036	2,420

Standard errors in parentheses ***p < 0.01, **p < 0.05, *p < 0.1

To cope with the impact of the pandemic at the household level in Pakistan, the estimations show that families primarily switched to low-quality food, increasing the degree of severity by 21.2%. This is true across the three models and is greater in urban areas (23.2%) than in rural areas (15.8%). For example, qualitative variations in staple foods such as rice and wheat are quite common. One can expect households to switch from commonly used full-grain high-quality (sabut) rice to short-grain broken (tota) low-quality rice, which is significantly cheaper. In combination with the food intake reduction of 14%, food security was severely impacted by 35%. In the second position, we estimate the marginal effect of bank loans or NGOs to be largest, even though it was the least adapted coping strategy in the debt burden category. However, it is consistently negative (− 16.7%; − 11.9%; − 17.0%). This implies that banking and NGO loans actively decreased the severity of the pandemic for Pakistani households, almost 5% more so for urban households.

According to the State Bank of Pakistan and its COVID-19 loan extension and restructuring package of debt relief, 96.92% of applications were approved by different banks under different loan categories, including microfinancing, which is undertaken mostly by NGOs in Pakistan [41]. Therefore, in line with this, our results also show a highly statistically significant marginal effect of loan deferment in the full and urban models. In addition, loans from employers with low statistical significance and a small marginal effect are also estimated for the full and rural models.

The next coping strategy with a marginal effect of 14.7% is taking out loans from relatives or friends, hence showing the significance of informal safety nets essential to the survival of households during the pandemic in many low- or middle-income countries. Similarly, financial community assistance as a gift has a statistically significant marginal effect of only 8.5%. In the energy poverty category, we discovered that rural households have been hit by electricity defaults, increasing the severity of the pandemic by 12%. On the other hand, electricity defaults had no effect on urban households. In addition, gas defaults were not statistically significant. It is somewhat intuitive to expect that rural households do not hold electricity as essential as urban households do, thus delaying payments and increasing the severity of the pandemic.^4^ Gas bills are relatively minor expenses in Pakistan because of their local extraction and because of available alternatives, such as wood and cow dung [59, 60].

In the essential category, there were no statistically significant results that led to school discontinuation or access to health. Only displacement in the full model and urban households accounted for 6.6% and 8.5%, respectively. As the schools were regularly conducted through several communication media, such as the internet (teleschool), radio and portable drives, displacement did not result in significant school dropout [61]. We provide further cross reference to this in Sect. "Substitution and heterogeneity analysis". In the capital flight category, the results suggest that household assets were sold and added to the severity of the pandemic, with marginal effects of 7.3% and 9.5% in the full model and urban model, respectively. On the other hand, usage of savings and investments is highly statistically significant for the rural model, with a marginal effect of 7.6%, but not for the other two models. Finally, consumption of stock seed for the next season is also predicted to be statistically significant at the 10% level for the full model. The adoption of these categories shows that while they did contribute to a minor increase in the severity of the pandemic, other productive assets, cattle and land, were not impacted by the pandemic. Our control variables reveal that young and above average income rural households may have been relatively severely impacted by a loss of income and a decrease in quality of life. A summary of our results on the basis of the severity of the strategy is provided in Table 5.

**Table 5:** Summary of results based on marginal effects

Full model	Rural Pakistan	Urban Pakistan
( +) Low quality food***	( +) Low quality food***	( +) Low quality food***
(−) Loan Banks***	( +) Loan relatives***	(-) Loan Banks***
( +) Loan relatives***	( +) Food reduction***	( +) Loan relatives***
( +) Food reduction***	( +) Electricity defaults***	( +) Food reduction***
( +) Community assistance***	(−) Loan Banks*	( +) Household Assets***
( +) Stock seed*	( +) Community assistance***	( +) Displacement**
( +) Loan deferment***	( +) Savings***	( +) Community assistance***
( +) Household Assets***	( +) Loan Employer*	( +) Loan deferment***
( +) Displacement**	( +) Young Households**	
( +) Electricity defaults***	( +) Above Average Income*	
( +) Loan Employer*		
( +) Young Households*		

***p < 0.01, **p < 0.05, *p < 0.1

To assess potential substitutions or trade-offs between coping strategies, we provide line wise results of our full model in Appendix 3, Tables 15 and 16.

### Substitution and heterogeneity analysis

We also attempt to disentangle the regional heterogeneity across the provincial territories of Pakistan, wherever necessary, and include the results in Appendix 2, which also serves as a further robustness check of our models. There are a few observed changes in the sizes of probabilities and their statistical significance. Primarily, bank loans are consistently estimated to be negative with an increase in their marginal effects. Note that with the addition of the community assistance variable, bank loans further decrease the severity of household impact by almost 7%. This implies that, combined with community assistance, household resilience through bank loans may have improved. Similar combined resilience can also be observed between food reduction and low-quality food variables. A change of almost 15% occurs in impact severity due to food reduction as soon as a household has an option to switch to low-quality food.

In the essential category, school dropout is initially statistically significant at the 10% level, with a 4% contribution to the severity of the household. However, with the possibility of selling productive assets, it becomes less statistically significant and eventually becomes insignificant when controlling for provincial regional effects, with the exception of KP. This implies that school dropouts were hedged by selling highly reliant productive assets (sewing machines, wheelbarrows, grain mills, agricultural tools, farm machinery, bicycles, cars, etc.). Additionally, a KP-wide phenomenon was observed but with a lower statistical significance. This is important because it provides an intertemporal understanding of the households: a short-term choice of occupational assets vs. long-term investment in human capital. Displacement also shows fluctuations in its statistical significance in the presence of other coping strategies. In the capital flight category, a substitution between productive assets and household assets can be observed. This approach is somewhat intuitive for predicting that households may prefer to sell their household assets (e.g., jewelry, furniture, or television) instead of selling their productive assets.

A similar substitution between selling productive cattle and stock seed is also highlighted by the results. Stock seed emerge as a consistent dominant coping strategy instead of selling productive cattle (e.g., productive female animals). Finally, we discovered that charity efforts aimed to cushion against the impact of the pandemic more so in KP than in other provinces. Regionally, our results revealed an active decrease in the severity of the pandemic at the household level across Punjab and Sindh but not in the KP and Balochistan regions. This is not surprising, as governmental assistance and regulatory efforts are disproportionately directed toward the densely populated regions of Punjab (approximately 55% of the population) and Sindh (approximately 25% of the population) [62].

## Discussion of results and implications

In the previous section, we presented our results with three models: a full model, a model focused on urban data and another model with only rural data. The outcomes of the research were roughly comparable across the whole model and the urban model, which is unexpected given that 62% of the overall population lives in rural settings, according to World Development Indicators [4]. The results can be attributed to two main reasons: 1) urban areas, though smaller in number, are hubs of economic activity (including health facilities, employment, investment and housing opportunities), and 2) urban migration is a major phenomenon in Pakistan, i.e., most urban areas have greater concentrations of population as villagers migrate to these areas in search of better economic and social opportunities [63]. Thus, the social and economic impacts of the pandemic may have been transmitted between urban and rural areas. Moreover, since the data were collected through electronic methods, they were more easily accessible in urban settings than in rural areas.

Unsurprisingly, food security was predicted as the major concern for households during the pandemic, and results highlighted that the key coping mechanism was either borrowing from banks or from relatives while reducing household expenditure. These results align with previous studies, which highlight food security as a critical concern and expenditure reduction as a coping mechanism for households during the pandemic [64, 65]. According to the estimations, food security remains a major problem, particularly for impoverished households in rural areas of the country. Moreover, individuals, especially those in urban areas, usually prefer loans from banks because microfinance options are relatively easy. Similarly, owing to strong cultural and social linkages, support from employers, family and relatives are predicted to remain significant coping mechanism for rural households, which is in line with previous findings on the role of family in dealing with the adverse effects of crisis [66, 67].

Charity (i.e., a sense of community) may also play a significant role in reducing the severity of the impact of COVID-19 and in coping with the shock. Religion plays a key role in the lives of Pakistanis citizens, and giving charity to people in need and desperate crisis in particular is strongly obligated and practiced. Literature studies have also pointed out the importance of charities in coping with poverty and pandemic [56, 68]. Evidence also suggests that external shocks increase a sense of community among people and that they are willing to help each other out (through charity or loans) to make survival possible, which are in line with the past findings [69–71]. Support for poor people through charities was a common practice throughout the country during the pandemic, which also complemented the voluntary behaviors of Pakistani citizens during the crisis.

On a broader level, external shocks do impact spending and saving patterns at the household level, as suggested by rational choice theory and findings of similar studies in the past [57, 72]. In middle-income countries, food security has become one of the primary factors affected by external shocks such as the pandemic. Most of the poor in middle-income nations are sensitive to income and price variations and thus adjust their spending patterns as soon as they observe an incoming shock. External shocks may worsen existing vulnerabilities, resulting in reduced access to food and increasing food insecurity among families.

Coping strategies may differ somewhat between urban and rural settings: rural areas have less access to financial institutions and therefore rely on either informal borrowing or selling of (nonproductive) assets. In comparison, people in urban areas may better able to cope with external shocks because of better accessibility to banks and financial institutions to hedge their losses. Similarly, we observed that the impact of external shocks and their reactions also differ across geographical locations. In areas with higher populations (urban areas) and more economic activity, the government are assumed to put in more effort and has more targeted policies in place than do less economically significant regions. This result is consistent with other studies, which highlight that government responses to external shocks often vary by geographical and economic significance [73, 74]. This strategy of the government has the drawback of further increasing regional inequalities. It is essential for the government to not implement a one-size-fits-all strategy in the country but rather work together with local governments and civil society organizations to develop a targeted approach for different parts of the country.

Analyzing the results from a trickledown approach, it can be observed that in times of external shocks, the trickle-down effect may not work as expected because individuals become increasingly risk averse as they burn through their savings and assets. In such times, it is advisable to opt for targeted strategies focusing on low- and middle-income populations to increase confidence in the economy as suggested in some literature works [70, 75].

Moreover, based on previous studies to cope with pandemics and help communities, policies on increased digitalization (including better internet infrastructure and increased digital literacy) and better access to financial institutions should be a focus for countries with high rural populations [76]. The results show that the rural population has limited confidence (or knowledge) in opportunities for formal lending, which support the findings of previous studies highlighting low financial literacy and accessibility barriers [77]. The government, in coordination with local NGOs and civil society organizations, needs to introduce campaigns to increase the understanding of the rural population about formal lending options. Moreover, it should also incentivize banks to encourage investment and lending in remote areas to ensure that the trickledown effect is felt in the poorest (or remotest) part of society [15, 75].

Our results may provide the starting point for recovery under further escalation and worsening of situations across the globe; as war wages progress, supply chains remain disrupted, and inflation continues to evaporate purchasing power. The recovery may remain in progress but seems hardened by these factors for households in countries such as Pakistan. Political instability, the role of international development and financial institutions, and the impact of climate change need further reconsideration to complete these multifaceted shocks.

## Conclusion

This study offers insightful information on the coping mechanisms used by Pakistani households to address the complex problems caused by the COVID-19 epidemic. The results highlight the relevance of food security combined with the usage of financial support as the main strategy used by households to lessen the impact on their income and show how commonplace nonfood decreases are. Furthermore, the small difference between urban and rural locations highlights the need for intervention measures that are targeted and customized to each area. Importantly, during times of crisis, the trickle-down effect may not follow the usual pattern; thus, its limits become apparent, emphasizing the significance of inclusive economic policies and social safety nets in mitigating the negative effects of external shocks on family wellbeing.

Although, this study provides key insights into household coping mechanisms during COVID-19, it is important to recognize the limitations. First, the research is based on data gathered during a specific time in 2020, which may not fully account for the pandemic's dynamics that are constantly evolving with time. Longitudinal studies are necessary to gain deeper knowledge of how families respond to pandemic waves (or other similar shocks) that are prolonged or recurrent, can enable in adapting inclusive policies to address future challenges. Second, we are cautiously optimistic that our results are transferable to other countries in the low- and lower-middle-income categories because the Pakistan Act seems to have common elements with several other countries, but more broader studies may account for deeper insights. For instance, even though bank loans were the only coping strategy that significantly reduced the likelihood of households being severely affected by COVID-19, it is still important to examine the larger socioeconomic and policy contexts that affect the efficacy of these strategies. Finally, future studies can explore the complex web of elements that affect family resilience, such as access to government assistance programs, healthcare, and education. As the world continues to grapple with the enduring effects of economic and geopolitical shocks, further research is needed to adapt and refine coping strategies to ensure the already hardened economic well-being of households in the face of evolving challenges.

## Data Availability

Data that supports the findings of this study are freely available online from the Pakistan Bureau of Statistics. (2020), Special Survey for Evaluating Socio-Economic Impact of COVID-19 on Wellbeing of People. Retrieved February 17, 2024, from Microdata (COVID-19) website: https://www.pbs.gov.pk/content/microdata-covid-19.

## Appendix 1

See Tables 6, 7, 8, 9, 10 and 11.

**Table 6:** Descriptive statistics

Variable	Obs	Mean	Std. Dev	Min	Max
AffectedHH	3456	0.280	0.449	0.000	1.000
Foodreduction	3456	0.698	0.459	0.000	1.000
Lowqualityfood	3456	0.754	0.431	0.000	1.000
Nonfoodreduction	3456	0.839	0.367	0.000	1.000
Savings	3456	0.723	0.448	0.000	1.000
Loanrelatives	3456	0.443	0.497	0.000	1.000
Loanemployer	3456	0.120	0.325	0.000	1.000
Loanbanks	3456	0.045	0.208	0.000	1.000
Communityassistance	3456	0.128	0.334	0.000	1.000
Loandeferment	3456	0.218	0.413	0.000	1.000
Schooldropout	3456	0.168	0.374	0.000	1.000
Electricitydefault	3456	0.404	0.491	0.000	1.000
Gasdefault	3456	0.269	0.443	0.000	1.000
Displacement	3456	0.035	0.184	0.000	1.000
Productiveassets	3456	0.057	0.232	0.000	1.000
HHassets	3456	0.077	0.266	0.000	1.000
Productivecattle	3456	0.083	0.276	0.000	1.000
Stockseed	3456	0.024	0.154	0.000	1.000
Soldland	3456	0.010	0.100	0.000	1.000
YoungHH	3456	0.503	0.500	0.000	1.000
MaledominatedHH	3456	0.679	0.467	0.000	1.000
EducatedHH	3456	0.364	0.481	0.000	1.000
AboveavgHHincome	3456	0.401	0.490	0.000	1.000
Remittances	3456	0.034	0.181	0.000	1.000
Charity	3456	0.401	0.490	0.000	1.000
RuralHH	3456	0.300	0.458	0.000	1.000
KP	3456	0.131	0.337	0.000	1.000
Punjab	3456	0.318	0.466	0.000	1.000
Sindh	3456	0.326	0.469	0.000	1.000
Balochistan	3456	0.120	0.325	0.000	1.000
Otherregion1	3456	1.000	0.000	1.000	1.000
Otherregion2	3456	1.000	0.000	1.000	1.000

**Table 7:** Descriptive statistics rural households

Variable	Obs	Mean	Std. Dev	Min	Max
AffectedHH	1036	0.268	0.443	0.000	1.000
Foodreduction	1036	0.712	0.453	0.000	1.000
Lowqualityfood	1036	0.764	0.425	0.000	1.000
Nonfoodreduction	1036	0.810	0.393	0.000	1.000
Savings	1036	0.618	0.486	0.000	1.000
Loanrelatives	1036	0.401	0.490	0.000	1.000
Loanemployer	1036	0.149	0.356	0.000	1.000
Loanbanks	1036	0.035	0.183	0.000	1.000
Communityassistance	1036	0.095	0.293	0.000	1.000
Loandeferment	1036	0.191	0.393	0.000	1.000
Schooldropout	1036	0.102	0.303	0.000	1.000
Electricitydefault	1036	0.375	0.484	0.000	1.000
Gasdefault	1036	0.179	0.383	0.000	1.000
Displacement	1036	0.030	0.170	0.000	1.000
Productiveassets	1036	0.059	0.236	0.000	1.000
HHassets	1036	0.058	0.234	0.000	1.000
Productivecattle	1036	0.172	0.377	0.000	1.000
Stockseed	1036	0.045	0.208	0.000	1.000
Soldland	1036	0.008	0.088	0.000	1.000
YoungHH	1036	0.481	0.500	0.000	1.000
MaledominatedHH	1036	0.656	0.475	0.000	1.000
EducatedHH	1036	0.346	0.476	0.000	1.000
AboveavgHHincome	1036	0.355	0.479	0.000	1.000
Remittances	1036	0.031	0.173	0.000	1.000
Charity	1036	0.384	0.487	0.000	1.000

**Table 8:** Descriptive statistics KPK

Variable	Obs	Mean	Std. Dev	Min	Max
AffectedHH	452	0.288	0.453	0.000	1.000
Foodreduction	452	0.631	0.483	0.000	1.000
Lowqualityfood	452	0.788	0.409	0.000	1.000
Nonfoodreduction	452	0.832	0.374	0.000	1.000
Savings	452	0.628	0.484	0.000	1.000
Loanrelatives	452	0.555	0.497	0.000	1.000
Loanemployer	452	0.069	0.253	0.000	1.000
Loanbanks	452	0.015	0.124	0.000	1.000
Communityassistance	452	0.086	0.281	0.000	1.000
Loandeferment	452	0.164	0.370	0.000	1.000
Schooldropout	452	0.033	0.179	0.000	1.000
Electricitydefault	452	0.378	0.486	0.000	1.000
Gasdefault	452	0.201	0.401	0.000	1.000
Displacement	452	0.024	0.154	0.000	1.000
Productiveassets	452	0.027	0.161	0.000	1.000
HHassets	452	0.073	0.260	0.000	1.000
Productivecattle	452	0.049	0.215	0.000	1.000
Stockseed	452	0.011	0.105	0.000	1.000
Soldland	452	0.011	0.105	0.000	1.000
YoungHH	452	0.451	0.498	0.000	1.000
MaledominatedHH	452	0.699	0.459	0.000	1.000
EducatedHH	452	0.341	0.474	0.000	1.000
AboveavgHHincome	452	0.347	0.477	0.000	1.000
Remittances	452	0.066	0.249	0.000	1.000
Charity	452	0.409	0.492	0.000	1.000

**Table 9:** Descriptive statistics Punjab

Variable	Obs	Mean	Std. Dev	Min	Max
AffectedHH	1100	0.172	0.377	0.000	1.000
Foodreduction	1100	0.790	0.407	0.000	1.000
Lowqualityfood	1100	0.785	0.411	0.000	1.000
Nonfoodreduction	1100	0.885	0.320	0.000	1.000
Savings	1100	0.801	0.399	0.000	1.000
Loanrelatives	1100	0.439	0.497	0.000	1.000
Loanemployer	1100	0.083	0.276	0.000	1.000
Loanbanks	1100	0.048	0.214	0.000	1.000
Communityassistance	1100	0.126	0.332	0.000	1.000
Loandeferment	1100	0.145	0.352	0.000	1.000
Schooldropout	1100	0.122	0.327	0.000	1.000
Electricitydefault	1100	0.353	0.478	0.000	1.000
Gasdefault	1100	0.235	0.424	0.000	1.000
Displacement	1100	0.043	0.202	0.000	1.000
Productiveassets	1100	0.062	0.241	0.000	1.000
HHassets	1100	0.091	0.288	0.000	1.000
Productivecattle	1100	0.081	0.273	0.000	1.000
Stockseed	1100	0.012	0.108	0.000	1.000
Soldland	1100	0.011	0.104	0.000	1.000
YoungHH	1100	0.559	0.497	0.000	1.000
MaledominatedHH	1100	0.677	0.468	0.000	1.000
EducatedHH	1100	0.337	0.473	0.000	1.000
AboveavgHHincome	1100	0.425	0.494	0.000	1.000
Remittances	1100	0.031	0.173	0.000	1.000
Charity	1100	0.325	0.469	0.000	1.000

**Table 10:** Descriptive statistics sindh

Variable	Obs	Mean	Std. Dev	Min	Max
AffectedHH	1127	0.322	0.467	0.000	1.000
Foodreduction	1127	0.721	0.449	0.000	1.000
Lowqualityfood	1127	0.770	0.421	0.000	1.000
Nonfoodreduction	1127	0.818	0.386	0.000	1.000
Savings	1127	0.688	0.464	0.000	1.000
Loanrelatives	1127	0.440	0.497	0.000	1.000
Loanemployer	1127	0.151	0.358	0.000	1.000
Loanbanks	1127	0.061	0.240	0.000	1.000
Communityassistance	1127	0.167	0.373	0.000	1.000
Loandeferment	1127	0.195	0.397	0.000	1.000
Schooldropout	1127	0.212	0.409	0.000	1.000
Electricitydefault	1127	0.465	0.499	0.000	1.000
Gasdefault	1127	0.384	0.487	0.000	1.000
Displacement	1127	0.035	0.183	0.000	1.000
Productiveassets	1127	0.070	0.255	0.000	1.000
HHassets	1127	0.080	0.271	0.000	1.000
Productivecattle	1127	0.088	0.283	0.000	1.000
Stockseed	1127	0.048	0.214	0.000	1.000
Soldland	1127	0.012	0.111	0.000	1.000
YoungHH	1127	0.489	0.500	0.000	1.000
MaledominatedHH	1127	0.681	0.466	0.000	1.000
EducatedHH	1127	0.391	0.488	0.000	1.000
AboveavgHHincome	1127	0.358	0.480	0.000	1.000
Remittances	1127	0.006	0.079	0.000	1.000
Charity	1127	0.492	0.500	0.000	1.000

**Table 11:** Descriptive statistics balochistan

Variable	Obs	Mean	Std. Dev	Min	Max
AffectedHH	414	0.432	0.496	0.000	1.000
Foodreduction	414	0.667	0.472	0.000	1.000
Lowqualityfood	414	0.775	0.418	0.000	1.000
Nonfoodreduction	414	0.857	0.350	0.000	1.000
Savings	414	0.749	0.434	0.000	1.000
Loanrelatives	414	0.481	0.500	0.000	1.000
Loanemployer	414	0.263	0.441	0.000	1.000
Loanbanks	414	0.036	0.187	0.000	1.000
Communityassistance	414	0.099	0.299	0.000	1.000
Loandeferment	414	0.423	0.495	0.000	1.000
Schooldropout	414	0.336	0.473	0.000	1.000
Electricitydefault	414	0.459	0.499	0.000	1.000
Gasdefault	414	0.316	0.466	0.000	1.000
Displacement	414	0.034	0.181	0.000	1.000
Productiveassets	414	0.068	0.251	0.000	1.000
HHassets	414	0.082	0.275	0.000	1.000
Productivecattle	414	0.092	0.289	0.000	1.000
Stockseed	414	0.007	0.085	0.000	1.000
Soldland	414	0.007	0.085	0.000	1.000
YoungHH	414	0.428	0.495	0.000	1.000
MaledominatedHH	414	0.688	0.464	0.000	1.000
EducatedHH	414	0.314	0.465	0.000	1.000
AboveavgHHincome	414	0.396	0.490	0.000	1.000
Remittances	414	0.002	0.049	0.000	1.000
Charity	414	0.360	0.481	0.000	1.000

## Appendix 2

See Tables 12, 13 and 14.

**Table 12:** Correlation matrix—full model

Variables	(1)	(2)	(3)	(4)	(5)	(6)	(7)	(8)	(9)	(10)	(11)	(12)	(13)	(14)	(15)	(16)	(17)	(18)	(19)	(20)	(21)	(22)	(23)	(24)	(25)
(1) AffectedHH	1.000																								
(2) Foodreduction	0.271	1.000																							
(3) Lowqualityfood	0.292	0.639	1.000																						
(4) Loanrelatives	0.313	0.245	0.300	1.000																					
(5) Loanemployer	0.171	0.142	0.145	0.218	1.000																				
(6) Loanbanks	0.034	0.104	0.086	0.147	0.390	1.000																			
(7)Communityassistance	0.182	0.114	0.116	0.203	0.222	0.320	1.000																		
(8) Loandeferment	0.261	0.098	0.097	0.236	0.238	0.113	0.244	1.000																	
(9) Electricitydefaultt	0.196	0.117	0.086	0.171	0.097	0.075	0.177	0.221	1.000																
(10) Gasdefault	0.146	0.082	0.041	0.154	0.112	0.103	0.166	0.158	0.659	1.000															
(11) Nonfoodreduction	0.178	0.332	0.471	0.274	0.108	0.073	0.109	0.081	0.004	-0.007	1.000														
(12) Schooldropout	0.188	0.147	0.127	0.129	0.194	0.092	0.164	0.349	0.263	0.245	0.083	1.000													
(13) Displacement	0.078	0.050	0.036	0.052	0.095	0.132	0.092	0.078	0.106	0.133	0.045	0.129	1.000												
(14) Productiveassets	0.128	0.094	0.080	0.112	0.167	0.120	0.123	0.118	0.092	0.138	0.043	0.157	0.157	1.000											
(15) Savings	0.082	0.112	0.157	0.140	0.016	0.014	0.055	0.095	-0.036	0.006	0.283	0.131	-0.001	0.046	1.000										
(16) HHassets	0.148	0.137	0.109	0.122	0.108	0.109	0.127	0.092	0.111	0.132	0.064	0.124	0.099	0.333	0.055	1.000									
(17) Productivecattle	0.108	0.092	0.086	0.085	0.145	0.050	0.058	0.050	0.024	-0.040	0.068	0.048	0.103	0.193	0.036	0.178	1.000								
(18) Stockseed	0.094	0.059	0.047	0.007	0.098	0.074	0.125	0.030	0.058	0.065	0.043	0.060	0.133	0.172	0.005	0.138	0.335	1.000							
(19) Soldland	0.027	-0.003	-0.023	0.003	0.061	0.075	0.065	0.023	0.046	0.063	-0.034	0.071	0.138	0.187	-0.021	0.188	0.116	0.247	1.000						
(20) YoungHH	-0.009	-0.031	-0.049	-0.032	-0.040	-0.011	-0.017	-0.017	-0.002	0.006	-0.042	0.009	0.007	-0.028	0.004	0.010	-0.034	-0.016	0.008	1.000					
(21) MaledominatedHH	0.011	0.012	-0.003	0.001	0.019	0.010	-0.017	-0.028	-0.012	-0.011	-0.011	-0.009	0.006	0.009	-0.001	0.000	0.002	0.000	-0.023	0.100	1.000				
(22) EducatedHH	-0.014	-0.047	-0.067	-0.017	-0.027	0.017	-0.002	0.002	-0.018	-0.031	-0.044	-0.007	-0.007	-0.030	-0.015	-0.019	-0.037	-0.037	0.002	0.072	-0.027	1.000			
(23)AboveavgHHincome	0.006	-0.018	-0.028	0.004	0.014	0.017	0.005	0.017	0.035	0.010	-0.047	0.019	0.014	-0.031	0.020	0.008	-0.010	-0.045	0.000	0.050	-0.004	0.091	1.000		
(24) Remittances	-0.003	-0.051	-0.012	-0.032	-0.049	-0.018	-0.029	-0.010	-0.050	-0.059	0.017	-0.033	0.017	-0.025	0.012	-0.012	0.037	0.002	-0.019	0.010	0.002	-0.002	0.039	1.000	
(25) Charity	0.061	0.007	0.018	0.046	0.033	0.020	0.049	0.047	0.018	0.039	0.040	0.059	0.002	0.056	0.038	0.050	0.022	0.059	0.017	-0.002	0.005	-0.020	-0.015	-0.055	1.000

**Table 13:** Correlation matrix—rural pakistan

Variables	(1)	(2)	(3)	(4)	(5)	(6)	(7)	(8)	(9)	(10)	(11)	(12)	(13)	(14)	(15)	(16)	(17)	(18)	(19)	(20)	(21)	(22)	(23)	(24)	(25)
(1) AffectedHH	1.000																								
(2) Foodreduction	0.250	1.000																							
(3) Lowqualityfood	0.275	0.647	1.000																						
(4) Loanrelatives	0.332	0.245	0.333	1.000																					
(5) Loanemployer	0.267	0.152	0.194	0.279	1.000																				
(6) Loanbanks	0.052	0.086	0.043	0.135	0.261	1.000																			
(7) Communityassistance	0.221	0.082	0.063	0.254	0.171	0.263	1.000																		
(8) Loandeferment	0.315	0.108	0.108	0.234	0.335	0.109	0.287	1.000																	
(9) Electricitydefaultt	0.219	-0.053	-0.044	0.135	0.040	0.016	0.097	0.196	1.000																
(10) Gasdefault	0.099	-0.160	-0.175	0.046	0.103	0.063	0.159	0.087	0.565	1.000															
(11) Nonfoodreduction	0.194	0.355	0.595	0.316	0.140	0.065	0.131	0.117	-0.097	-0.198	1.000														
(12) Schooldropout	0.263	0.123	0.127	0.153	0.271	0.127	0.217	0.403	0.199	0.225	0.107	1.000													
(13) Displacement	0.073	0.024	0.017	0.041	0.102	0.152	0.156	0.059	0.110	0.214	0.056	0.165	1.000												
(14) Productiveassets	0.182	0.114	0.129	0.164	0.264	0.154	0.199	0.129	0.111	0.226	0.090	0.294	0.245	1.000											
(15) Savings	0.131	0.141	0.261	0.258	0.038	-0.013	0.091	0.099	-0.112	-0.121	0.398	0.108	-0.002	0.087	1.000										
(16) HHassets	0.130	0.112	0.118	0.168	0.140	0.133	0.174	0.027	0.158	0.187	0.089	0.216	0.199	0.394	0.101	1.000									
(17) Productivecattle	0.146	0.137	0.114	0.181	0.155	0.095	0.106	0.091	0.059	-0.012	0.136	0.100	0.115	0.212	0.132	0.249	1.000								
(18) Stockseed	0.119	0.087	0.066	0.030	0.118	0.136	0.231	0.036	0.099	0.141	0.082	0.125	0.207	0.162	0.066	0.105	0.294	1.000							
(19) Soldland	0.046	-0.017	-0.029	0.018	0.087	0.164	0.122	0.069	0.068	0.074	-0.042	0.116	0.243	0.165	-0.021	0.167	0.077	0.193	1.000						
(20) YoungHH	0.006	-0.059	-0.044	-0.065	-0.065	-0.024	-0.001	0.034	0.016	0.031	-0.041	0.000	0.012	-0.052	-0.030	-0.032	-0.075	-0.033	-0.041	1.000					
(21) MaledominatedHH	0.016	0.057	-0.009	-0.010	-0.012	-0.040	-0.030	-0.005	-0.048	-0.029	-0.014	-0.084	-0.028	-0.009	-0.025	-0.038	-0.042	-0.008	-0.029	0.098	1.000				
(22) EducatedHH	0.036	-0.067	-0.037	-0.014	-0.024	0.017	0.001	0.034	0.023	0.006	-0.025	0.043	0.027	-0.035	-0.017	-0.041	-0.024	-0.012	0.052	0.040	0.000	1.000			
(23) AboveavgHHincome	0.069	-0.014	-0.021	-0.022	0.030	0.013	-0.047	0.024	0.062	0.017	-0.021	-0.051	-0.012	-0.057	0.094	-0.037	0.009	-0.075	-0.019	0.053	-0.024	0.097	1.000		
(24) Remittances	-0.033	-0.010	0.020	-0.021	-0.059	-0.003	-0.020	-0.044	-0.046	-0.069	0.015	-0.042	-0.031	-0.021	0.037	-0.020	0.037	-0.039	-0.016	0.029	0.012	-0.036	0.031	1.000	
(25) Charity	0.086	-0.024	-0.025	0.059	0.016	0.034	0.111	0.065	0.047	0.124	0.034	0.093	0.036	0.106	0.050	0.051	0.040	0.114	0.066	0.023	0.012	-0.015	-0.010	-0.015	1.000

**Table 14:** Correlation matrix—urban Pakistan

Variables	(1)	(2)	(3)	(4)	(5)	(6)	(7)	(8)	(9)	(10)	(11)	(12)	(13)	(14)	(15)	(16)	(17)	(18)	(19)	(20)	(21)	(22)	(23)	(24)	(25)
(1) AffectedHH	1.000																								
(2) Foodreduction	0.280	1.000																							
(3) Lowqualityfood	0.299	0.636	1.000																						
(4) Loanrelatives	0.305	0.248	0.289	1.000																					
(5) Loanemployer	0.127	0.136	0.121	0.196	1.000																				
(6) Loanbanks	0.028	0.112	0.102	0.149	0.453	1.000																			
(7) Communityassistance	0.168	0.128	0.137	0.181	0.252	0.336	1.000																		
(8) Loandeferment	0.239	0.095	0.094	0.235	0.200	0.113	0.227	1.000																	
(9) Electricitydefaultt	0.187	0.188	0.141	0.184	0.129	0.095	0.203	0.229	1.000																
(10) Gasdefault	0.162	0.171	0.119	0.185	0.130	0.110	0.160	0.176	0.696	1.000															
(11) Nonfoodreduction	0.170	0.324	0.417	0.252	0.096	0.074	0.096	0.062	0.047	0.058	1.000														
(12) Schooldropout	0.164	0.160	0.132	0.116	0.182	0.078	0.142	0.331	0.282	0.235	0.068	1.000													
(13) Displacement	0.079	0.060	0.043	0.055	0.094	0.125	0.070	0.084	0.104	0.106	0.039	0.118	1.000												
(14) Productiveassets	0.105	0.085	0.058	0.091	0.118	0.109	0.096	0.114	0.085	0.110	0.021	0.115	0.123	1.000											
(15) Savings	0.057	0.105	0.115	0.074	0.017	0.019	0.028	0.085	-0.009	0.028	0.214	0.121	-0.005	0.028	1.000										
(16) HHassets	0.154	0.148	0.108	0.103	0.101	0.100	0.110	0.112	0.092	0.109	0.052	0.093	0.066	0.312	0.027	1.000									
(17) Productivecattle	0.099	0.062	0.070	0.045	0.125	0.042	0.061	0.043	0.016	-0.014	0.039	0.070	0.116	0.199	0.020	0.179	1.000								
(18) Stockseed	0.086	0.039	0.033	0.000	0.076	0.049	0.084	0.036	0.038	0.048	0.023	0.049	0.100	0.189	-0.019	0.180	0.365	1.000							
(19) Soldland	0.020	0.003	-0.020	-0.004	0.052	0.048	0.047	0.007	0.038	0.057	-0.033	0.057	0.104	0.196	-0.025	0.194	0.168	0.307	1.000						
(20) YoungHH	−0.017	−0.018	−0.050	−0.021	−0.025	−0.008	−0.025	−0.039	−0.011	−0.009	−0.045	0.008	0.004	−0.017	0.014	0.023	0.006	0.000	0.025	1.000					
(21) MaledominatedHH	0.009	−0.007	0.000	0.003	0.037	0.027	−0.015	−0.039	0.002	−0.010	−0.011	0.011	0.019	0.017	0.003	0.012	0.050	0.011	−0.022	0.100	1.000				
(22) EducatedHH	−0.036	−0.038	−0.079	−0.021	−0.027	0.016	−0.005	−0.012	−0.036	−0.049	−0.055	−0.026	−0.020	−0.028	−0.019	−0.013	−0.042	−0.054	−0.017	0.084	−0.040	1.000			
(23) AboveavgHHincome	−0.022	−0.018	−0.030	0.010	0.012	0.016	0.017	0.011	0.021	−0.004	−0.064	0.032	0.023	−0.019	−0.030	0.020	−0.002	−0.018	0.005	0.047	0.001	0.087	1.000		
(24) Remittances	0.009	−0.067	−0.024	−0.037	−0.044	−0.023	−0.033	0.002	−0.052	−0.059	0.016	−0.032	0.034	−0.027	−0.001	−0.010	0.046	0.031	−0.020	0.002	−0.003	0.011	0.042	1.000	
(25) Charity	0.051	0.021	0.036	0.039	0.043	0.014	0.025	0.039	0.004	0.006	0.041	0.046	−0.012	0.035	0.028	0.049	0.020	0.027	0.000	−0.014	0.002	−0.023	−0.019	−0.072	1.000

## Appendix 3

See Tables 15 and 16.

**Table 15:** Logit estimates—full model for substitution effects

	(1)	(2)	(3)	(4)	(5)	(6)	(7)	(8)	(9)	(10)	(11)	(12)
(1)	1.678***	0.877***	0.802***	0.773***	0.795***	0.771***	0.770***	0.733***	0.732***	0.728***	0.714***	0.713***
	(0.114)	(0.129)	(0.132)	(0.133)	(0.133)	(0.134)	(0.136)	(0.137)	(0.137)	(0.137)	(0.137)	(0.137)
(2)		1.779***	1.490***	1.469***	1.467***	1.468***	1.490***	1.494***	1.495***	1.426***	1.416***	1.420***
		(0.179)	(0.182)	(0.183)	(0.183)	(0.184)	(0.186)	(0.186)	(0.186)	(0.192)	(0.192)	(0.192)
(3)			1.133***	1.070***	1.095***	1.036***	0.939***	0.890***	0.889***	0.871***	0.880***	0.883***
			(0.0850)	(0.0863)	(0.0866)	(0.0875)	(0.0891)	(0.0900)	(0.0901)	(0.0909)	(0.0911)	(0.0912)
(4)				0.513***	0.705***	0.660***	0.453***	0.463***	0.462***	0.465***	0.438***	0.437***
				(0.115)	(0.126)	(0.128)	(0.131)	(0.132)	(0.132)	(0.132)	(0.133)	(0.133)
(5)					− 0.752***	− 1.170***	− 1.068***	− 1.077***	− 1.079***	− 1.082***	− 1.079***	− 1.127***
					(0.201)	(0.219)	(0.220)	(0.220)	(0.221)	(0.221)	(0.222)	(0.224)
(6)						0.798***	0.599***	0.530***	0.529***	0.522***	0.516***	0.514***
						(0.122)	(0.127)	(0.128)	(0.128)	(0.128)	(0.128)	(0.128)
(7)							0.904***	0.808***	0.808***	0.812***	0.746***	0.745***
							(0.0990)	(0.101)	(0.101)	(0.101)	(0.105)	(0.105)
(8)								0.472***	0.462***	0.466***	0.444***	0.442***
								(0.0884)	(0.111)	(0.111)	(0.112)	(0.112)
(9)									0.0174	0.00965	− 0.0219	− 0.0396
									(0.120)	(0.120)	(0.121)	(0.121)
(10)										0.231	0.238	0.231
										(0.175)	(0.175)	(0.175)
(11)											0.259**	0.242**
											(0.115)	(0.115)
(12)												0.443**
												(0.214)
(13)												
(14)												
(15)												
(16)												
(17)												
(18)												
(19)												
(20)												
(21)												
(22)												
(23)												
(24)												
(25)												
Const	− 2.256***	− 3.155***	− 3.448***	− 3.445***	− 3.464***	− 3.509***	− 3.653***	− 3.775***	− 3.775***	− 3.911***	− 3.911***	− 3.916***
	(0.106)	(0.163)	(0.167)	(0.167)	(0.168)	(0.168)	(0.171)	(0.173)	(0.173)	(0.204)	(0.204)	(0.204)
N	3456	3456	3456	3456	3456	3456	3456	3456	3456	3456	3456	3456
r2_p	0.0708	0.102	0.148	0.153	0.156	0.167	0.187	0.194	0.194	0.194	0.195	0.196
chi2	290.0	418.6	605.3	625.1	639.6	682.0	764.9	793.1	793.1	794.9	800.0	804.2
ll	− 1903	− 1838	− 1745	− 1735	− 1728	− 1707	− 1665	− 1651	− 1651	− 1650	− 1648	− 1646

	(13)	(14)	(15)	(16)	(17)	(18)	(19)	(20)	(21)	(22)	(23)	(24)	(25)
(1)	0.706***	0.706***	0.680***	0.673***	0.670***	0.669***	0.668***	0.667***	0.667***	0.667***	0.673***	0.676***	0.677***
	(0.137)	(0.137)	(0.137)	(0.138)	(0.138)	(0.138)	(0.138)	(0.138)	(0.138)	(0.138)	(0.138)	(0.138)	(0.138)
(2)	1.417***	1.417***	1.417***	1.417***	1.422***	1.419***	1.425***	1.426***	1.433***	1.434***	1.433***	1.444***	1.446***
	(0.192)	(0.192)	(0.192)	(0.192)	(0.192)	(0.192)	(0.193)	(0.193)	(0.193)	(0.193)	(0.193)	(0.193)	(0.193)
(3)	0.877***	0.875***	0.867***	0.864***	0.883***	0.882***	0.883***	0.883***	0.882***	0.882***	0.885***	0.882***	0.881***
	(0.0913)	(0.0914)	(0.0916)	(0.0916)	(0.0921)	(0.0921)	(0.0921)	(0.0922)	(0.0922)	(0.0922)	(0.0923)	(0.0923)	(0.0923)
(4)	0.407***	0.409***	0.417***	0.386***	0.385***	0.385***	0.389***	0.384***	0.387***	0.385***	0.392***	0.391***	0.398***
	(0.134)	(0.134)	(0.134)	(0.135)	(0.136)	(0.136)	(0.136)	(0.136)	(0.136)	(0.136)	(0.136)	(0.136)	(0.137)
(5)	− 1.134***	− 1.134***	− 1.164***	− 1.148***	− 1.151***	− 1.149***	− 1.150***	− 1.154***	− 1.161***	− 1.161***	− 1.164***	− 1.163***	− 1.168***
	(0.225)	(0.225)	(0.227)	(0.228)	(0.229)	(0.228)	(0.229)	(0.229)	(0.229)	(0.229)	(0.229)	(0.229)	(0.229)
(6)	0.502***	0.502***	0.486***	0.486***	0.460***	0.460***	0.461***	0.463***	0.463***	0.463***	0.466***	0.462***	0.458***
	(0.129)	(0.129)	(0.129)	(0.129)	(0.130)	(0.130)	(0.130)	(0.130)	(0.130)	(0.130)	(0.130)	(0.130)	(0.130)
(7)	0.739***	0.738***	0.740***	0.746***	0.753***	0.752***	0.754***	0.757***	0.757***	0.757***	0.756***	0.753***	0.753***
	(0.106)	(0.106)	(0.106)	(0.106)	(0.106)	(0.106)	(0.106)	(0.106)	(0.106)	(0.106)	(0.106)	(0.106)	(0.106)
(8)	0.450***	0.452***	0.449***	0.437***	0.440***	0.440***	0.441***	0.442***	0.443***	0.442***	0.443***	0.453***	0.457***
	(0.112)	(0.112)	(0.112)	(0.112)	(0.112)	(0.112)	(0.112)	(0.112)	(0.112)	(0.112)	(0.112)	(0.113)	(0.113)
(9)	− 0.0640	− 0.0658	− 0.0800	− 0.0460	− 0.0592	− 0.0585	− 0.0588	− 0.0577	− 0.0525	− 0.0522	− 0.0495	− 0.0566	− 0.0667
	(0.122)	(0.122)	(0.122)	(0.123)	(0.124)	(0.124)	(0.124)	(0.124)	(0.124)	(0.124)	(0.124)	(0.124)	(0.126)
(10)	0.232	0.226	0.228	0.221	0.210	0.209	0.212	0.214	0.221	0.223	0.218	0.215	0.215
	(0.175)	(0.177)	(0.178)	(0.178)	(0.178)	(0.178)	(0.178)	(0.178)	(0.178)	(0.178)	(0.178)	(0.178)	(0.178)
(11)	0.225*	0.222*	0.214*	0.218*	0.218*	0.220*	0.217*	0.217*	0.217*	0.216*	0.218*	0.207*	0.202*
	(0.116)	(0.116)	(0.117)	(0.117)	(0.117)	(0.117)	(0.117)	(0.117)	(0.117)	(0.117)	(0.117)	(0.117)	(0.118)
(12)	0.384*	0.385*	0.373*	0.337	0.307	0.313	0.312	0.311	0.309	0.307	0.298	0.306	0.304
	(0.217)	(0.217)	(0.218)	(0.220)	(0.221)	(0.222)	(0.221)	(0.222)	(0.221)	(0.222)	(0.222)	(0.222)	(0.223)
(13)	0.409**	0.408**	0.257	0.201	0.168	0.175	0.180	0.178	0.181	0.184	0.189	0.179	0.181
	(0.172)	(0.172)	(0.180)	(0.182)	(0.183)	(0.184)	(0.184)	(0.184)	(0.184)	(0.184)	(0.184)	(0.184)	(0.184)
(14)		0.0219	0.0207	0.0134	0.0199	0.0203	0.0172	0.0169	0.0150	0.0147	0.0138	0.00757	0.00298
		(0.104)	(0.104)	(0.104)	(0.104)	(0.104)	(0.104)	(0.104)	(0.104)	(0.104)	(0.104)	(0.104)	(0.105)
(15)			0.449***	0.409***	0.398***	0.406***	0.401***	0.401***	0.401***	0.400***	0.399**	0.388**	0.384**
			(0.153)	(0.154)	(0.154)	(0.155)	(0.155)	(0.155)	(0.155)	(0.155)	(0.155)	(0.155)	(0.156)
(16)				0.341**	0.224	0.225	0.229	0.230	0.236	0.236	0.227	0.233	0.250
				(0.148)	(0.156)	(0.156)	(0.156)	(0.156)	(0.156)	(0.156)	(0.157)	(0.157)	(0.160)
(17)					0.693**	0.715**	0.719**	0.718**	0.723**	0.729**	0.727**	0.692**	0.698**
					(0.279)	(0.283)	(0.283)	(0.283)	(0.284)	(0.284)	(0.285)	(0.285)	(0.286)
(18)						− 0.240	− 0.251	− 0.240	− 0.241	− 0.243	− 0.232	− 0.208	− 0.218
						(0.500)	(0.500)	(0.499)	(0.500)	(0.500)	(0.500)	(0.501)	(0.502)
(19)							0.0785	0.0702	0.0650	0.0634	0.0631	0.0645	0.0643
							(0.0866)	(0.0870)	(0.0871)	(0.0872)	(0.0872)	(0.0873)	(0.0873)
(20)								0.0973	0.0993	0.0995	0.0989	0.0978	0.0960
								(0.0934)	(0.0934)	(0.0934)	(0.0934)	(0.0935)	(0.0936)
(21)									0.0950	0.0910	0.0916	0.0951	0.0946
									(0.0908)	(0.0912)	(0.0913)	(0.0913)	(0.0913)
(22)										0.0387	0.0367	0.0371	0.0350
										(0.0889)	(0.0889)	(0.0890)	(0.0891)
(23)											0.292	0.322	0.319
											(0.240)	(0.241)	(0.241)
(24)												0.204**	0.203**
												(0.0880)	(0.0880)
(25)													− 0.0523
													(0.102)
Const	− 3.915***	− 3.925***	− 3.923***	− 3.929***	− 3.935***	− 3.932***	− 3.977***	− 4.041***	− 4.088***	− 4.103***	− 4.114***	− 4.202***	− 4.182***
	(0.204)	(0.210)	(0.210)	(0.210)	(0.211)	(0.211)	(0.217)	(0.226)	(0.231)	(0.233)	(0.234)	(0.238)	(0.241)
N	3456	3456	3456	3456	3456	3456	3456	3456	3456	3456	3456	3456	3456
r2_p	0.198	0.198	0.200	0.201	0.203	0.203	0.203	0.203	0.203	0.203	0.204	0.205	0.205
chi2	809.9	809.9	818.5	823.7	829.8	830.1	830.9	832.0	833.1	833.3	834.7	840.1	840.3
ll	− 1643	− 1643	− 1638	− 1636	− 1633	− 1633	− 1632	− 1632	− 1631	− 1631	− 1630	− 1628	− 1627

**Table 16:** Logit marginal effects—full model for substitution effects

	(1)	(2)	(3)	(4)	(5)	(6)	(7)	(8)	(9)	(10)	(11)	(12)
(1)	0.313***	0.159***	0.136***	0.130***	0.133***	0.127***	0.123***	0.116***	0.116***	0.115***	0.113***	0.112***
	(0.0195)	(0.0229)	(0.0221)	(0.0221)	(0.0220)	(0.0217)	(0.0215)	(0.0214)	(0.0214)	(0.0214)	(0.0213)	(0.0213)
(2)		0.323***	0.253***	0.248***	0.246***	0.242***	0.238***	0.236***	0.236***	0.225***	0.223***	0.224***
		(0.0315)	(0.0304)	(0.0303)	(0.0302)	(0.0298)	(0.0292)	(0.0289)	(0.0289)	(0.0299)	(0.0298)	(0.0298)
(3)			0.193***	0.180***	0.184***	0.171***	0.150***	0.141***	0.140***	0.138***	0.139***	0.139***
			(0.0129)	(0.0132)	(0.0132)	(0.0133)	(0.0134)	(0.0135)	(0.0135)	(0.0136)	(0.0136)	(0.0136)
(4)				0.0865***	0.118***	0.109***	0.0723***	0.0731***	0.0731***	0.0734***	0.0691***	0.0689***
				(0.0191)	(0.0207)	(0.0209)	(0.0208)	(0.0207)	(0.0207)	(0.0207)	(0.0208)	(0.0208)
(5)					− 0.126***	− 0.193***	− 0.171***	− 0.170***	− 0.171***	− 0.171***	− 0.170***	− 0.178***
					(0.0335)	(0.0356)	(0.0347)	(0.0344)	(0.0344)	(0.0344)	(0.0345)	(0.0348)
(6)						0.132***	0.0958***	0.0837***	0.0836***	0.0825***	0.0814***	0.0810***
						(0.0197)	(0.0200)	(0.0200)	(0.0201)	(0.0201)	(0.0200)	(0.0200)
(7)							0.145***	0.128***	0.128***	0.128***	0.118***	0.117***
							(0.0150)	(0.0154)	(0.0154)	(0.0154)	(0.0161)	(0.0161)
(8)								0.0745***	0.0730***	0.0736***	0.0701***	0.0696***
								(0.0137)	(0.0174)	(0.0174)	(0.0174)	(0.0174)
(9)									0.00274	0.00152	− 0.00345	− 0.00623
									(0.0189)	(0.0190)	(0.0191)	(0.0191)
(10)										0.0366	0.0375	0.0363
										(0.0276)	(0.0276)	(0.0276)
(11)											0.0409**	0.0381**
											(0.0181)	(0.0181)
(12)												0.0698**
												(0.0337)
(13)												
(14)												
(15)												
(16)												
(17)												
(18)												
(19)												
(20)												
(21)												
(22)												
(23)												
(24)												
(25)												
N	3456	3456	3456	3456	3456	3456	3456	3456	3456	3456	3456	3456

	(13)	(14)	(15)	(16)	(17)	(18)	(19)	(20)	(21)	(22)	(23)	(24)	(25)
(1)	0.111***	0.111***	0.106***	0.105***	0.105***	0.104***	0.104***	0.104***	0.104***	0.104***	0.105***	0.105***	0.105***
	(0.0213)	(0.0213)	(0.0213)	(0.0213)	(0.0212)	(0.0212)	(0.0212)	(0.0212)	(0.0212)	(0.0212)	(0.0212)	(0.0212)	(0.0212)
(2)	0.223***	0.223***	0.222***	0.221***	0.222***	0.221***	0.222***	0.222***	0.223***	0.223***	0.223***	0.224***	0.225***
	(0.0297)	(0.0297)	(0.0296)	(0.0296)	(0.0296)	(0.0296)	(0.0296)	(0.0296)	(0.0296)	(0.0296)	(0.0296)	(0.0295)	(0.0295)
(3)	0.138***	0.138***	0.136***	0.135***	0.138***	0.138***	0.138***	0.138***	0.137***	0.137***	0.138***	0.137***	0.137***
	(0.0136)	(0.0136)	(0.0136)	(0.0136)	(0.0137)	(0.0137)	(0.0137)	(0.0137)	(0.0136)	(0.0136)	(0.0136)	(0.0136)	(0.0136)
(4)	0.0640***	0.0643***	0.0653***	0.0603***	0.0601***	0.0601***	0.0606***	0.0598***	0.0602***	0.0600***	0.0611***	0.0607***	0.0618***
	(0.0209)	(0.0209)	(0.0209)	(0.0211)	(0.0211)	(0.0211)	(0.0211)	(0.0211)	(0.0211)	(0.0211)	(0.0211)	(0.0211)	(0.0212)
(5)	− 0.178***	− 0.178***	− 0.182***	− 0.179***	− 0.179***	− 0.179***	− 0.179***	− 0.180***	− 0.181***	− 0.181***	− 0.181***	− 0.181***	− 0.181***
	(0.0349)	(0.0349)	(0.0350)	(0.0351)	(0.0351)	(0.0351)	(0.0351)	(0.0351)	(0.0351)	(0.0351)	(0.0351)	(0.0351)	(0.0351)
(6)	0.0788***	0.0788***	0.0761***	0.0759***	0.0717***	0.0718***	0.0718***	0.0721***	0.0721***	0.0720***	0.0726***	0.0718***	0.0712***
	(0.0200)	(0.0200)	(0.0201)	(0.0200)	(0.0201)	(0.0201)	(0.0201)	(0.0201)	(0.0201)	(0.0201)	(0.0201)	(0.0201)	(0.0201)
(7)	0.116***	0.116***	0.116***	0.117***	0.117***	0.117***	0.118***	0.118***	0.118***	0.118***	0.118***	0.117***	0.117***
	(0.0161)	(0.0161)	(0.0161)	(0.0160)	(0.0160)	(0.0160)	(0.0160)	(0.0160)	(0.0160)	(0.0160)	(0.0160)	(0.0160)	(0.0160)
(8)	0.0707***	0.0710***	0.0704***	0.0682***	0.0686***	0.0686***	0.0688***	0.0688***	0.0690***	0.0688***	0.0690***	0.0704***	0.0710***
	(0.0174)	(0.0174)	(0.0174)	(0.0174)	(0.0174)	(0.0174)	(0.0174)	(0.0174)	(0.0174)	(0.0174)	(0.0174)	(0.0173)	(0.0174)
(9)	− 0.0100	− 0.0103	− 0.0125	− 0.00718	− 0.00923	− 0.00913	− 0.00916	− 0.00899	− 0.00817	− 0.00813	− 0.00770	− 0.00880	− 0.0104
	(0.0191)	(0.0192)	(0.0192)	(0.0193)	(0.0193)	(0.0193)	(0.0193)	(0.0193)	(0.0193)	(0.0193)	(0.0193)	(0.0193)	(0.0195)
(10)	0.0364	0.0355	0.0358	0.0346	0.0327	0.0326	0.0330	0.0333	0.0344	0.0347	0.0340	0.0334	0.0335
	(0.0275)	(0.0279)	(0.0278)	(0.0277)	(0.0277)	(0.0277)	(0.0277)	(0.0277)	(0.0278)	(0.0278)	(0.0277)	(0.0277)	(0.0277)
(11)	0.0353*	0.0348*	0.0335*	0.0341*	0.0340*	0.0343*	0.0338*	0.0339*	0.0337*	0.0336*	0.0339*	0.0322*	0.0313*
	(0.0181)	(0.0182)	(0.0182)	(0.0182)	(0.0182)	(0.0182)	(0.0182)	(0.0182)	(0.0182)	(0.0182)	(0.0182)	(0.0182)	(0.0182)
(12)	0.0603*	0.0605*	0.0583*	0.0527	0.0478	0.0488	0.0486	0.0484	0.0482	0.0478	0.0463	0.0475	0.0472
	(0.0340)	(0.0340)	(0.0341)	(0.0343)	(0.0345)	(0.0345)	(0.0345)	(0.0345)	(0.0345)	(0.0345)	(0.0345)	(0.0345)	(0.0345)
(13)	0.0642**	0.0641**	0.0403	0.0314	0.0261	0.0273	0.0281	0.0277	0.0281	0.0287	0.0293	0.0278	0.0281
	(0.0269)	(0.0269)	(0.0282)	(0.0285)	(0.0285)	(0.0286)	(0.0286)	(0.0286)	(0.0286)	(0.0286)	(0.0286)	(0.0286)	(0.0286)
(14)		0.00344	0.00325	0.00210	0.00310	0.00317	0.00269	0.00263	0.00233	0.00229	0.00214	0.00118	0.000462
		(0.0163)	(0.0163)	(0.0163)	(0.0163)	(0.0163)	(0.0163)	(0.0163)	(0.0163)	(0.0163)	(0.0163)	(0.0162)	(0.0163)
(15)			0.0702***	0.0638***	0.0620***	0.0633***	0.0625***	0.0625***	0.0624***	0.0623***	0.0621***	0.0603**	0.0597**
			(0.0238)	(0.0240)	(0.0240)	(0.0241)	(0.0241)	(0.0241)	(0.0241)	(0.0241)	(0.0241)	(0.0241)	(0.0241)
(16)				0.0533**	0.0350	0.0351	0.0357	0.0359	0.0368	0.0367	0.0354	0.0362	0.0388
				(0.0231)	(0.0243)	(0.0243)	(0.0243)	(0.0243)	(0.0243)	(0.0243)	(0.0243)	(0.0243)	(0.0249)
(17)					0.108**	0.112**	0.112**	0.112**	0.113**	0.114***	0.113**	0.107**	0.108**
					(0.0434)	(0.0440)	(0.0440)	(0.0440)	(0.0440)	(0.0441)	(0.0441)	(0.0442)	(0.0443)
(18)						− 0.0375	− 0.0392	− 0.0374	− 0.0376	− 0.0378	− 0.0361	− 0.0324	− 0.0339
						(0.0779)	(0.0779)	(0.0778)	(0.0779)	(0.0779)	(0.0779)	(0.0779)	(0.0779)
(19)							0.0122	0.0109	0.0101	0.00988	0.00982	0.0100	0.00999
							(0.0135)	(0.0135)	(0.0136)	(0.0136)	(0.0136)	(0.0136)	(0.0136)
(20)								0.0152	0.0155	0.0155	0.0154	0.0152	0.0149
								(0.0145)	(0.0145)	(0.0145)	(0.0145)	(0.0145)	(0.0145)
(21)									0.0148	0.0142	0.0143	0.0148	0.0147
									(0.0141)	(0.0142)	(0.0142)	(0.0142)	(0.0142)
(22)										0.00603	0.00571	0.00577	0.00544
										(0.0138)	(0.0138)	(0.0138)	(0.0138)
(23)											0.0455	0.0501	0.0496
											(0.0373)	(0.0374)	(0.0374)
(24)												0.0317**	0.0316**
												(0.0136)	(0.0136)
(25)													− 0.00813
													(0.0159)
N	3456	3456	3456	3456	3456	3456	3456	3456	3456	3456	3456	3456	3456

## Footnotes

1 We do not engage in the debate of Rational Choice Theory and its plausibility.

2 We use the fitstat package of STATA Corp [47].

3 Payments made regularly under Islamic law on income and assets held by individuals.

4 It has also been personal experiences of the authors (a & b) that alternative to electricity is usual to be employed in the form of oil lamps, candles, and alike. Pakistani households are prepared for such electricity outages due to consistent issue of load shedding. Water, and means of cooking are rather more essentials in such situations.
